# A Genomic‐Based Workflow for eDNA Assay Development for a Critically Endangered Turtle, *Myuchelys georgesi*

**DOI:** 10.1002/ece3.70798

**Published:** 2025-01-08

**Authors:** Holly V. Nelson, Arthur Georges, Katherine A. Farquharson, Elspeth A. McLennan, Jane L. DeGabriel, Katherine Belov, Carolyn J. Hogg

**Affiliations:** ^1^ School of Life and Environmental Sciences The University of Sydney Sydney New South Wales Australia; ^2^ Institute for Applied Ecology University of Canberra Bruce Australian Capital Territory Australia; ^3^ Australian Research Council Centre of Excellence for Innovations in Peptide and Protein Science The University of Sydney Sydney New South Wales Australia; ^4^ NSW Department of Climate Change, The Environment, Energy and Water Parramatta New South Wales Australia

**Keywords:** Bellinger River turtle, eDNA, genomic data, *Myuchelys georgesi*, reference genome

## Abstract

Environmental DNA (eDNA) analysis has become a popular conservation tool for detecting rare and elusive species. eDNA assays typically target mitochondrial DNA (mtDNA) due to its high copy number per cell and its ability to persist in the environment longer than nuclear DNA. Consequently, the development of eDNA assays has relied on mitochondrial reference sequences available in online databases, or in cases where such data are unavailable, de novo DNA extraction and sequencing of mtDNA. In this study, we designed eDNA primers for the critically endangered Bellinger River turtle ( *Myuchelys georgesi* ) using a bioinformatically assembled mitochondrial genome (mitogenome) derived from a reference genome. We confirmed the accuracy of this assembled mitogenome by comparing it to a Sanger‐sequenced mitogenome of the same species, and no base pair mismatches were detected. Using the bioinformatically extracted mitogenome, we designed two 20 bp primers that target a 152‐base‐pair‐long fragment of the cytochrome oxidase 1 (CO1) gene and a 186‐base‐pair‐long fragment of the cytochrome B (CytB) gene. Both primers were successfully validated *in silico*, *in vitro*, and *in situ*.

## Graphical abstract

We designed eDNA primers for the critically endangered Bellinger River turtle ( *Myuchelys georgesi* ) using a bioinformatically assembled mitochondrial genome (mitogenome) derived from a reference genome. We validated these eDNA primers *in silico*, *in vitro* and *in situ*.

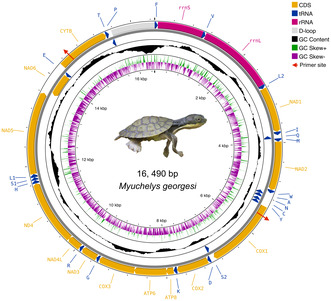

## 1 Introduction

In recent years, conservation geneticists have made substantial progress in understanding how to apply genetic data to conservation actions for threatened species (Hohenlohe, Funk, and Rajora 2021). The prevalence of cost‐effective, non‐invasive molecular tools like environmental DNA (eDNA) assays have become increasingly common in detecting invasive species, assessing community diversity across various spatial scales, and monitoring rare or cryptic species (Rees et al. 2014; Ardura et al. 2015; Ruppert, Kline, and Rahman 2019; Lam, Sung, and Fong 2022). eDNA refers to extra organismal genetic material the comprises of molecules that have been shed into the environment by decaying bodies, leaves, blood, pollen, seeds, urine, faeces, skin, hairs and other types of organismal material (Freeland 2017), that can be extracted from environmental samples such as soil, water and air (Barnes et al. 2014; Rees et al. 2014). The presence of eDNA can be detected using DNA metabarcoding for detection of entire communities or species‐specific primers or assays to detect a target species (Mauvisseau et al. 2019; Lopes et al. 2021; Valdivia‐Carrillo et al. 2021). eDNA assays commonly target and amplify a short fragment of mitochondrial DNA (mtDNA) through polymerase chain reaction (PCR). mtDNA is commonly targeted as it is highly abundant in cells and can persist in environments longer than nuclear DNA (nuDNA) (Wilcox et al. 2016; Bylemans et al. 2018).

Species‐specific eDNA marker development relies on the availability of mtDNA sequences in online databases, as demonstrated in recent studies on diamondback terrapins ( *Malaclemys terrapin* ) and red eared slider turtles (*Trachemys script elegans*) (Fields et al. 2024); reef sharks ( *Carcharhinus amblyrhynchos* ) (Dunn et al. 2023); and Atlantic wolf‐fish ( *Anarhichas lupus* ) (Chevrinais and Parent 2023). For species without publicly available mtDNA sequences, sequencing is required to facilitate marker development. Conventional methods for generating mtDNA sequence data have involved tissue acquisition, DNA extraction, designing universal primers, or primers of a closely related species, long‐range polymerase chain reactions (PCRs), shotgun sequencing, followed by bioinformatic assembly (Kundu et al. 2020; Chen et al. 2021; Tessler et al. 2023). The advent of high‐throughput parallel sequencing (HTS), reductions in sequencing costs and lower input DNA requirements, as well as improved bioinformatic pipelines, have given rise to the genomics era where traditional genetic approaches are being replaced by whole‐genome approaches to conservation genetic research (Satam et al. 2023). While genomic data alone have no direct impact on conservation outcomes, they provide a foundational blueprint that that can be harnessed by geneticists and conservationists for a range of downstream applications (Hogg et al. 2022). These can include; aiding in the identification of genetic variants for population genetic analyses (Brandies et al. 2019); investigations into functionally important genetic variation such as immune genes (Peel et al. 2022), development of PCR primers and recently *in silico* extraction of complete mitochondrial genomes (hereafter ‘mitogenomes’) (Meng et al. 2019; Uliano‐Silva, Nunes, and Krasheninnikova 2021).

The Bellinger River turtle ( *Myuchelys georgesi* ) is a species of short‐necked turtle (Family Chelidae) and is one of two turtle species that is listed as Critically Endangered in Australia under the Environmental Protection and Biodiversity Conservation Act 1999 (Commonwealth of Australia 1999). The species has a current known distribution that is restricted to 60 km of the Bellinger River and a short section of its main tributary, the Kalang River, in north‐eastern New South Wales (NSW), Australia (Cann et al. 2015). However, the species has not been recorded in the Kalang since 2007 (Georges et al. 2011). *Myuchelys georgesi* is a rare and cryptic species that has adapted to up‐stream regions of the Bellinger River, preferring deeper waterholes surrounded by bedrock making them difficult to survey using conventional diving and trapping methods (Spencer et al. 2014). In 2015, a novel nidovirus outbreak resulted in the estimated death of more than 90% of individuals, further contributing to the species' rarity (Zhang et al. 2018; Chessman et al. 2020). The species also faces threats from competition with another locally occurring species, the Murray River turtle ( *Emydura macquarii* ). Implementation of eDNA analyses in both known and data deficient areas of the catchment (e.g., Kalang River) is currently listed in the species Conservation Action Plan to inform survey site selection (R. Jakob‐Hoff et al., unpublished), yet no such tool currently exists.

Given the growing application of both eDNA and genomic data in conservation management, we used a PacBio HiFi reference genome to develop species‐specific eDNA markers for *M. georgesi* . We also provide comprehensive methodologies and visual workflow for other threatened species, with reference genomes or genomic data, which would benefit from an eDNA assay.

## 2 Methods

### 2.1 Mitogenome Assembly

**FIGURE 1:**
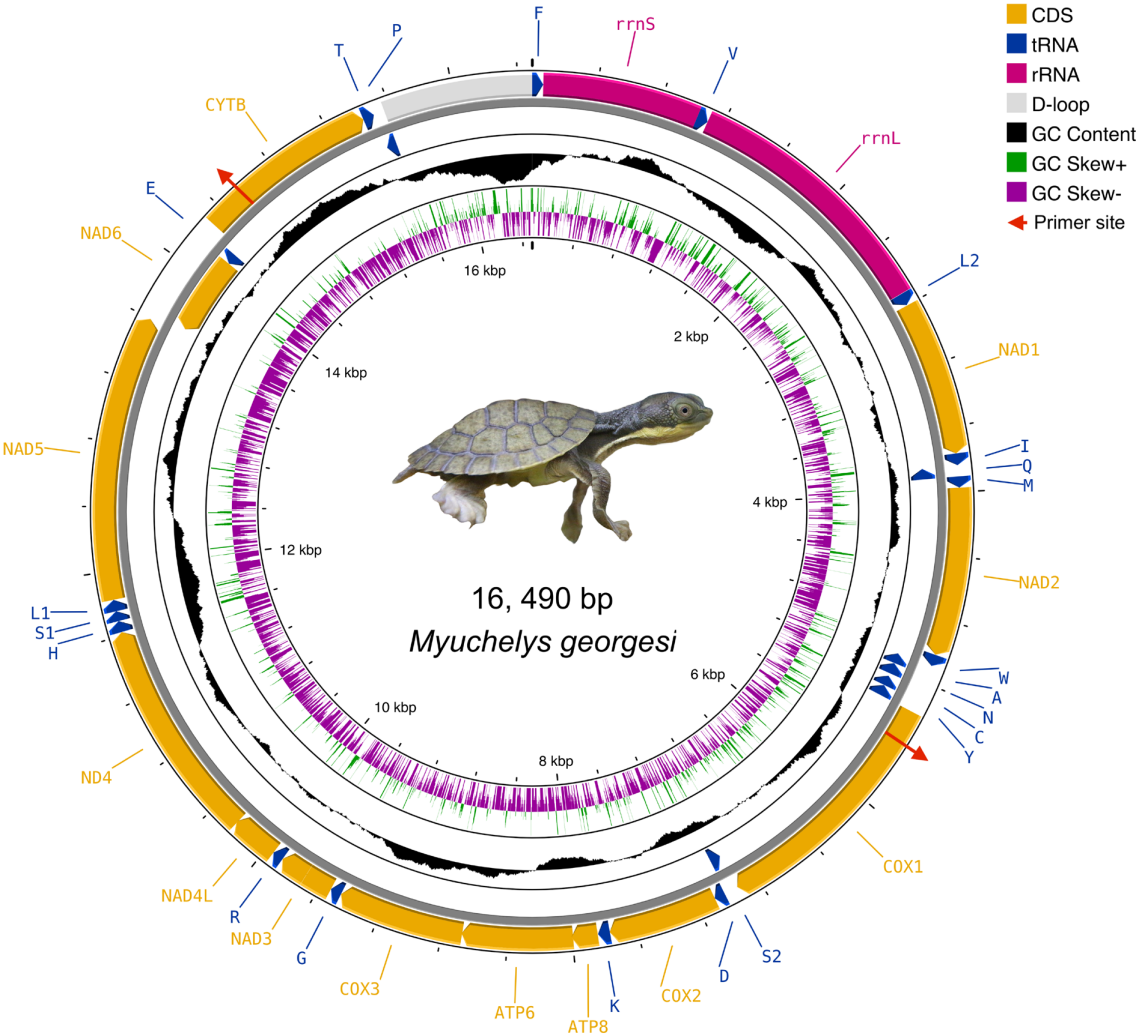
The mitochondrial genome of *M. georgesi* extracted using MitoHiFi (Uliano‐Silva, Nunes, and Krasheninnikova 2021). tRNAs are labelled according to their single‐letter abbreviation. Arrows indicate direction of gene transcription. Protein coding genes are shown in yellow, rRNA genes in pink, tRNA genes in blue and the 920 bp non‐coding region between P and F in white. The GC‐skew depicting the deviation from the average in the complete mitogenome is depicted in green (positive) and maroon (negative), and the GC content is depicted in black. Figure generated using the Proksee (https://proksee.ca). *M. georgesi* juvenile image credit of Paul Fahy.

We previously assembled a chromosome‐level reference genome for *M. georgesi* using PacBio High Fidelity (HiFi) (CA, USA) sequencing (Nelson et al. 2024). HiFi sequencing is a type of long‐read data that is generated by circular consensus sequencing (CCS). Raw CCS reads can be as long as 15,000–20,000 base pairs, allowing full‐length mitogenome sequences to be captured within a single read. To generate HiFi sequence data, high molecular weight DNA was extracted from the heart tissue of a male *M. georgesi* using the Nanobind Tissue Big DNA kit following the manufacturer's protocol (Circulomics, Pacific Biosciences, CA, USA). PacBio HiFi Single‐Molecule Real‐Time (SMRT) bell libraries were sequenced at the Australian Genome Research Facility (Brisbane, Australia). The HiFi genome was assembled using Hifiasm v.0.16.0 (Cheng et al. 2021). To obtain a complete mitogenome (i.e., the entire mitochondrial DNA), we bioinformatically extracted the mtDNA sequence from the HiFi genome fasta file (a text‐based file format containing nucleotide sequences) using MitoHifi v2 (Uliano‐Silva, Nunes, and Krasheninnikova 2021). The ‐c flag was used to identify and annotate the mitogenome from genome scaffolds, rather than assembling it from raw reads with the ‐r flag. MitoHiFi also requires a mitochondrial reference sequence as input in either fasta or GenBank format (e.g., https://www.ncbi.nlm.nih.gov/genbank/samplerecord/). MitoHiFi provides an internal script (findMitoReference.py) that can be used to find and download the most closely related mitogenome for the species of interest. For this study, we manually obtained reference sequences from the NCBI for the Green Sea Turtle ( *Chelonia mydas* ) (NC_000886) (Kumazawa and Nishida 1999), Murray River Turtle ( *Emydura macquarii* ) (NC_041302.1) (unpublished) and a previously Sanger sequenced *M. georgesi* mitogenome (NC_042474.1) (unpublished). These sequences were used to evaluate whether levels of divergence between reference and target species affected assembly quality. The mitogenome was visualised using Proksee (Grant et al. 2023) (Figure 1). To confirm efficacy of the bioinformatically extracted mitogenome, we used MEGA11 (Tamura, Stecher, and Kumar 2021) to align the assembly to a Sanger sequenced *M. georgesi* mitogenome for structural comparison and mismatches between sequences (NC_042474.1).

### 2.2 Species‐Specific Primer Development and Validation

Using the annotated fasta file output by MitoHiFi, we located genetic sequences labelled ‘CO1’ and ‘CytB’ and used the complete sequence (Figures S1 and S2) as input into Primer3Plus v3.3.0 (Untergasser et al. 2012) to design forward and reverse primer sequences with 0 base pair mismatches with the CO1 and CytB gene sequences. These genes were used as they are known to be highly variable among closely related species providing greater specificity for species‐specific eDNA assays compared to mitochondrial genes with lower inter‐specific variation (Moritz, Dowling, and Brown 1987; Meyer 1994; Johns and Avise 1998; Hebert, Ratnasingham, and de Waard 2003). Forward and reverse primers output by Primer3Plus were individually input into OligoAnalyzer (https://sg.idtdna.com/calc/analyzer) for quality checks using the hairpin and homodimer options to ensure efficiency and sensitivity of primer binding. To ensure primer stability and minimise the likelihood of hairpin structure formation (when complementary base‐pair sequences create a loop), we used a ΔG (Gibbs free energy change) threshold of −4.5 kcal/mol. For homodimers (annealing of identical primer sequences) we ensured primers had no more than three complementary bases. The melting temperature (*T*_*m*_) for all primer sequences fell between 59.6°C and 60.1°C. Final primer pairs are provided in Table 1 and Table S2. The specificity and sensitivity of primer sets were evaluated at three stages: *in silico*, *in vitro*, and *in situ*.

**TABLE 1:** Primers designed (CytB and CO1) and used (12S) in this study for amplification of *M. georgesi* mitochondrial eDNA. *T*_*m*_ melting temperature.

Gene	Name	Forward/Reverse	Nucleotide sequence	Primer length (bp)	Amplicon size (bp)	*T*_*m*_ (°C)
CytB	MG_CB	Forward	AATCTCCCACATCCAACGAG	20	186	59.9
		Reverse	ATGCGGTGGCTATGACTAGG			60.1
CO1	MG_C1	Forward	ACATTGGCACCCTCTACCTG	20	152	60
		Reverse	AATTAAGGCGTGGGCTGTAA			59.6
12S	12Sv5	Forward	TAGAACAGGCTCCTCTAG	18	~100	Riaz et al. (2011)
		Reverse	TTAGATACCCCACTATGC			

### 2.3 *In Silico* Validation

To confirm specificity *in silico*, the alignment search tool Basic Local Alignment Search Tool (BLAST) was used to confirm percent of sequence similarity with other species (https://blast.ncbi.nlm.nih.gov/). To visually confirm specificity and optimal primer design against another locally occurring species ( *E. macquarii* ) we used MEGA v11 to align both our assembled and *E. macquarii* mitogenomes (NC_041302.1) and ensured 2–3 mismatches between primer design sequences (de Brauwer et al. 2022b). Custom primer sets were ordered using ThermoFisher Scientific (MA, USA) custom DNA oligos synthesis service.

### 2.4 *In Vitro* Validation

To evaluate specificity and amplification efficiency *in vitro*, we carried out tests using tissue‐derived DNA from *M. georgesi* and *E. macquarii* using conventional PCR (Figure S2). Heart tissue belonging to a female *M. georgesi* that required medical euthanasia in 2021 (C10031) was flash frozen at −80°C at Taronga Zoo and stored at −80°C at the University of Sydney. *E. macquarii* skin tissue was acquired from the trailing webbing of the hindfoot of a wild individual in 2015 (UC<Aus>AA063724) and stored at −80°C in the University of Canberra Wildlife Tissue Collection (GenBank UC<Aus>). To prevent contamination during lab procedures, equipment was sterilised in an autoclave and benchtops cleaned with 80% ethanol. DNA (Table S3) was extracted using the Qiagen DNeasy Mini Kit (Qiagen, Germany) following the manufacturer's protocol, except for a final elution in 100 μL buffer AE (Qiagen). Quality (fragmentation) and concentration of DNA were assessed using a combination of a Nanodrop 2000 Spectrophotometer (ThermoFisher Scientific) and 1.5% agarose/TBE gel electrophoresis stained with SYBR safe (Life Technologies), alongside a 1 kb size standard (Bioline) and run for 55 min at 100 V. Samples yielding high concentrations of DNA were used for subsequent PCR amplification assays.

For PCR set‐up, 0.25 μM of CytB and CO1 forward and reverse primers were used. A quantity of 0.25 μM of 12Sv5F/12Sv5R universal vertebrate primers was used as a positive control by amplifying a ~100‐bp fragment of the V5 loop of the 12S mitochondrial gene (Riaz et al. 2011). The final PCR reaction consisted of 3 μL of *M. georgesi* DNA template or negative extraction control ( *E. macquarii* DNA template, ddH_2_O), 25 μL of Bioline MyTaq Mix (Bioline, UK), 2.5 μM of forward and reverse primers (either 12Sv5, CytB or CO1), and 17 μL of nuclease free water to make a total volume of 50 μL.

Real‐time PCR cycling was carried out on a T100 Thermal Cycler (BioRad). Cycling conditions were 10 min for enzyme activation at 95°C, 35 cycles of denaturation at 95°C for 30 s, annealing at 50°C for 30 s, extension at 72°C for 30 s and a final extension at 72°C for 10 min. Amplification was confirmed using 1.5% agarose/TBE gel electrophoresis stained with SYBR safe (Life Technologies), alongside a 1 kb size standard (Bioline) and run for 55 min at 100 V. Bands were visualised under ultraviolet light using a ChemiDoc XRS + system (BioRad) and images were analysed with ImageLab (BioRad).

### 2.5 *In Situ* Validation

*M. georgesi* eDNA water samples (positive controls) were obtained from three 4000 L, closed‐system tanks at Symbio Wildlife Park in Helensburgh, Australia, each housing four or five animals. From each tank, two 500 mL water samples were collected. For negative controls, we collected two 500 mL water samples from a 2000 L pond containing four *E. macquarii* and two Eastern long‐necked turtles (*Chelondina longicollis*). We transported the water samples on ice and stored them briefly at −2°C before filtering within 1–2 h of collection. Negative control samples were handled and stored separately to prevent contamination.

A 47 mm Whatman membrane filter paper with a pore size of 0.45 μm was dampened with deionised water before the 500 mL water samples were filtered through. The filtration system included a 50 mm Büchner funnel, adaptor, 500 mL Büchner flask, rubber tubing and a diaphragm pump (KNF, CA, USA). The filter papers were then placed in individual resealable bags and frozen at −80°C prior to DNA extraction the following day.

eDNA extractions were conducted on samples (Table S4) using Qiagen's DNeasy Blood & Tissue Kit (Qiagen, Germany). DNA extraction followed the protocol of Renshaw et al. (2015) with minor adjustments. Briefly, each filter paper was halved and finely cut before being placed in separate 2 mL screw‐cap tubes. Volumes of 540 μL buffer ATL and 60 μL (rather than the recommended 180 and 20 μL, respectively) of Proteinase K were added to submerse each half filter and incubated at 65°C for 1 h. Following lysis, the paper was tightly pressed to the bottom of the tube, and supernatant transferred to a new 2 mL screw‐cap tube. Volumes of 630 μL Buffer AL and 630 μL of ethanol were added and mixed thoroughly with a vortex. The lysates from each half were then combined by passing the mixtures through the same DNeasy Mini spin column, resulting in six rounds of centrifugation and discarded flow‐through. Total eDNA was rinsed with 500 μL of AW1 and AW2 solutions respectively and eluted in 100 μL buffer AE (Qiagen). eDNA concentration was quantified using a Nanodrop 2000 Spectrophotometer (ThermoFisher Scientific). All eDNA extractions were placed in a freezer (−20°C) for 12 h until PCR analysis.

Following our *in vitro* validation protocol, 0.25 μM of CytB and CO1 forward and reverse primers were used for PCR set‐up. A quantity of 0.25 μM of 12Sv5F/12Sv5R universal vertebrate primers was used as a positive control. PCR mixes consisted of 3 μL of *M. georgesi* eDNA template or a negative extraction control ( *E. macquarii* and *C. longicollis* eDNA template or ddH_2_O), 25 μL of Bioline MyTaq Mix (Bioline, UK), 2.5 μM of forward and reverse primers, and 17 μL of nuclease free water to make a total volume of 50 μL. Real‐time PCR cycling, agarose gel electrophoresis, and image analysis was conducted using the same methods described in *in vitro* validation section above.

## 3 Results

### 3.1 *Myuchelys georgesi* Mitogenome

The complete mitochondrial sequence was extracted from scaffold 9 of our reference assembly and yielded a complete length of 16,490 bp (Figure 1). The same mitogenome was assembled when the Green Sea Turtle, Murray River Turtle and Bellinger River Turtle mitogenomes were used as reference sequences, confirming that varying levels of divergence between reference input and target species does not affect final assembly quality. The size and structure of the mitochondrial genome is comparable to other chelid turtles (Fielder et al. 2012; Zhang et al. 2017), which includes 37 genes consisting of 22 transfer RNA (tRNA) genes, 13 protein coding genes, 2 ribosomal RNA (rRNA) genes, plus a non‐coding region (CR). Additional details can be found in the Supporting Information.

Visual alignment of the bioinformatically assembled mitogenome to the Sanger sequenced *M. georgesi* mitogenome (NC_042474.1) using MEGA v11 showed a 100% sequence identity match, confirming efficacy of the *in silico*‐based mitogenome.

### 3.2 Primer Design and Validation

**FIGURE 2:**
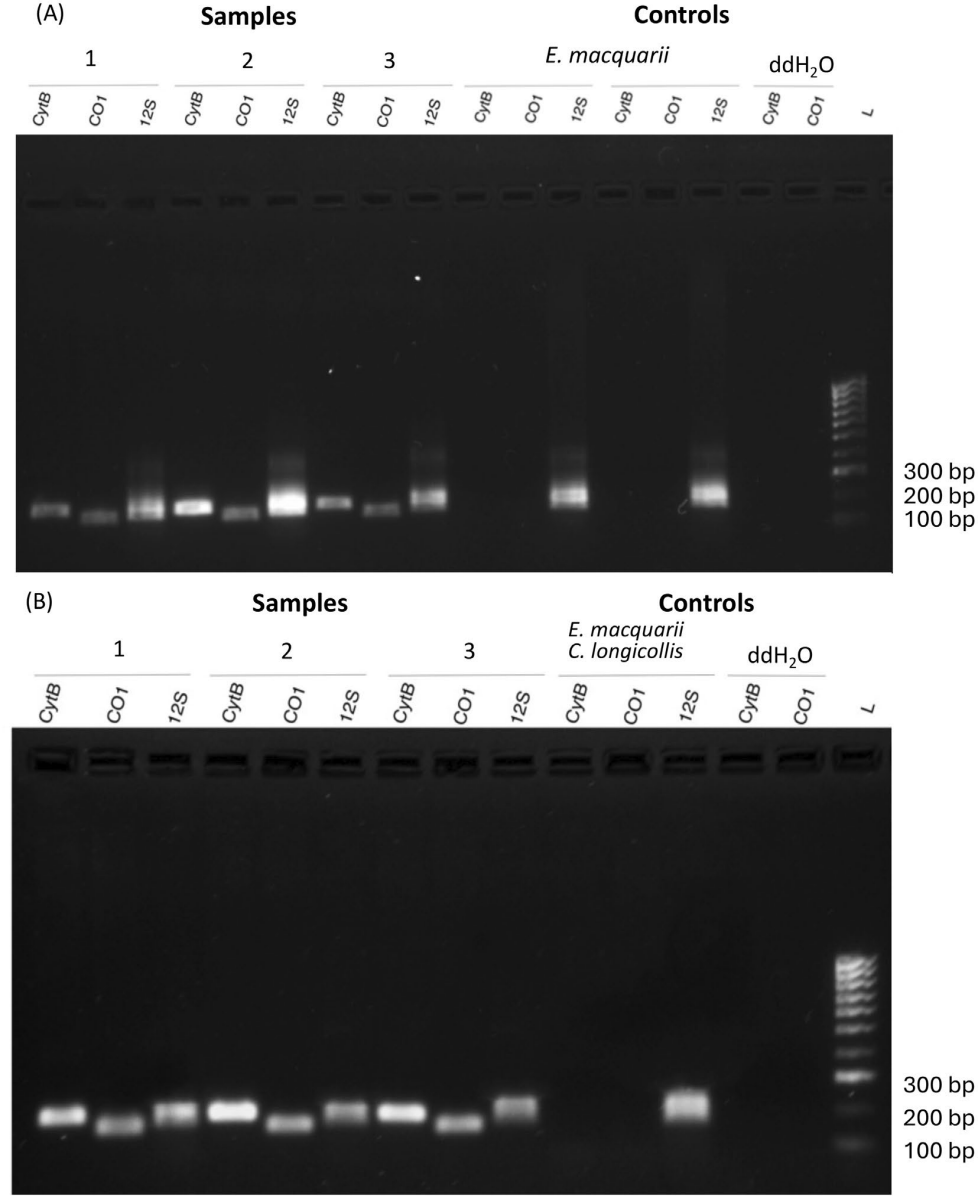
1.5% agarose gel and TBE stained with SYBR safe, showing (A) *in vitro* amplicon products of tissue derived DNA for Bellinger River Turtle ( *Myuchelys georgesi* ) with CytB, CO1 and 12Sv5 control (lanes 1–9), Murray River Turtle ( *Emydura macquarii* ) with CytB, CO1 and 12Sv5 control (lanes 10–15), and ddH_2_O with CytB and CO1 (16–17). (B) *In situ* amplicon products of tank water derived eDNA for Bellinger River Turtle ( *Myuchelys georgesi* ) with CytB, CO1 and 12Sv5 control (lanes 1–9), Murray River Turtle ( *Emydura macquarii* ) and Eastern long‐necked Turtle (*Chelondina longicollis*) with CytB, CO1 and 12Sv5 control (lanes 10–12), and ddH_2_O with CytB and CO1 (lanes 13–14).

*In silico* primer assessment found greater species‐specificity of the CO1 primers compared to CytB as BLAST results returned lower percentage identity with other species. Both CO1 and CytB primers successfully amplified *M. georgesi* tissue samples (Figure 2A; lanes 1–2, 4–5, 7–8). Both sets of primers showed no amplification on *E. macquarii* tissue (Figure 2A; lanes 10–11 and 13–14), confirming the species‐specificity of the primers against the other locally occurring species. The 12Sv5 positive control amplified across both species, indicating the presence of mitochondrial DNA in the tissues (Figure 2A; lanes 3, 6, 9, 12 and 15), while no amplification was observed for the ddH2O negative controls (Figure 2A; lanes 16–17). *In situ* evaluation showed PCR products for both primers successfully amplified *M. georgesi* eDNA collected on cellulose ester filters from tank water (Figure 2B; lanes 1–2, 4–5, 7–8). Primer sets did not amplify eDNA from tank water containing *E. macquarii* or *C. longicollis* (Figure 2B; lanes 10–11), confirming species‐specificity of primers against other locally occurring species. Amplification of the positive 12Sv5 control across tank water confirmed the presence of mtDNA in all samples (Figure 2B; lanes 3, 6, 9 and 12) while no amplification was observed for the ddH2O negative control (Figure 2B; lanes 13–14).

## 4 Discussion

We developed the first eDNA markers for detection of *M. georgesi* using an existing long‐read PacBio HiFi reference genome. The 100% sequence identity match between the Sanger sequenced and bioinformatically assembled mitogenome (NC_042474.1) and successful amplification of mtDNA across *in silico*, *in vitro* and *in situ* validations highlights the efficacy of genomic data‐derived mitogenome assemblies, without the need for targeted mitochondrial DNA tissue extraction and sequencing.

**FIGURE 3:**
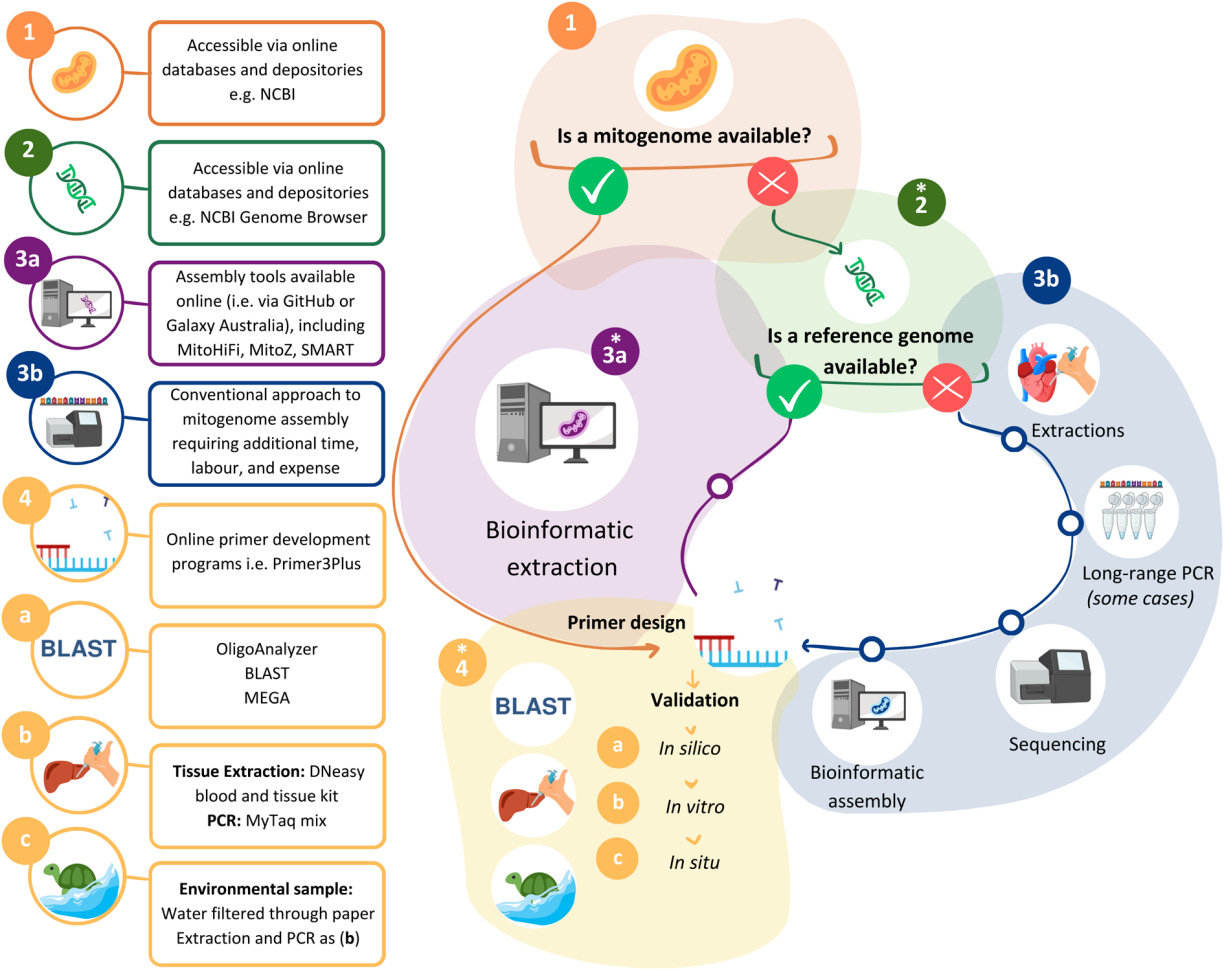
Reference genome derived eDNA assay workflow used for species‐specific primer development in *Myuchelys georgesi* . NCBI National Center for Biotechnology Information, BLAST Basic Local Alignment Search Tool, MEGA Molecular Evolutionary Genetic Analysis, PCR Polymerase Chain Reaction. Steps undertaken in this study are denoted by *. Image created using Biorender.com and Canva.com.

We provide comprehensive methodologies of our workflow for other taxa that may benefit from this approach (Figure 3). Conventional approaches rely on the availability of mitochondrial sequence data from online databases or de novo extraction, sequencing and assembly when sequence data is not available (Schmidt et al. 2016; Zhang et al. 2017; Kundu et al. 2019, 2020; Frandsen, Figueroa, and George 2020; Chen et al. 2021). For conservation programs with genomic resources but lacking mitochondrial sequence data, this approach offers an avenue for developing a widely used conservation genetic tool.

When developing a species‐specific eDNA assay, it is essential to have DNA sequence information unique to your target organism. The most efficient approach is identifying if relevant sequence data is available in online repositories (Figure 3: step 1) such as the National Centre for Biotechnology Information (NCBI), the Barcode of Life Data System (BOLD), and the European Molecular Biology Laboratory online repositories. The Sanger sequenced *M. georgesi* mitogenome used as our positive control for the bioinformatic mitogenome extraction was obtained from the NCBI database using ‘ *Myuchelys georgesi* mitochondrion’ as search terms. When relevant mitochondrial sequence data are not available in online repositories or if gene regions are missing for species or taxa (Freeland 2017; Nordstrom et al. 2022), genomic data (reference genome or raw HTS) provides an *in silico* alternative (Figure 3: step 2). For example, the availability of a reference genome has allowed for bioinformatic extraction of the mitogenomes for several cryptic and threatened species lacking mitochondrial sequence data including the Kroombit tinker frog ( *Taudactylus pleione* ) (Farquharson et al. 2023), blue‐tailed skink (*Cryptoblepharus egeriae*) (Dodge et al. 2023), Lister's gecko ( *Lepidodactylus listeri* ) (Dodge et al. 2023), and southern stuttering barred frog ( *Mixophyes balbus* ) (Tang et al. 2024), providing capacity for the development of species‐specific eDNA assays in the future. Although our approach leverages PacBio HiFi sequencing data and MitoHiFi mitochondrial genome assembly program for bioinformatic extraction (Figure 3: Step 3a), a suite of bioinformatic tools are available for extraction and assembly of mitogenomes from a range of HTS data types (Table 2). Additionally, some of these tools can take raw HTS sequencing data as input and do not require a reference genome.

**TABLE 2:** Bioinformatic tools for complete mitochondrial genome assembly using next generation sequencing (NGS) data (including whole genomes) for downstream eDNA assay development.

Name	Data input described	Reference
MitoHiFi	PacBio high fidelity (HiFi) (CCS) WGS data	Uliano‐Silva, Nunes, and Krasheninnikova (2021)
PMAT	PacBio high fidelity (HiFi) (CCS) WGS data	Bi et al. (2024)
SMART	Low‐coverage WGS	Alqahtani and Măndoiu (2020)
MitoZ	Short WGS raw reads	Meng et al. (2019)
Norgal	Short WGS raw reads	Al‐Nakeeb, Petersen, and Sicheritz‐Pontén (2017)
MITObim	Short WGS raw reads	Hahn, Bachmann, and Chevreux (2013)

Abbreviations: CCS, close consensus sequencing; CLR, continuous long reads; WGS, whole genome sequencing.

If mitochondrial or genomic sequence data does not exist (Figure 3: Step 1 and 2), conventional approaches involving acquisition of genetic material; DNA extraction; sequencing; and assembly are needed to undertake species' assay design (Figure 3: Step 3b). Although targeted mitochondrial sequencing may be effective when programs have limited funds available (Schmidt, Thia, and Hoffmann 2024), these approaches often require substantial time and resources to undertake so likely cost the same as whole genome sequencing when labour costs are accounted for. For example, completion of the existing *M. georgesi* mitogenome following the methods of Zhang et al. (2017) used Sanger sequencing and long‐range PCR, took 12 weeks to complete, costing $15,000 AUD in labour and $1500 in lab consumables (Arthur Georges pers. comm., 2024). By‐passing these steps, when genomic data is available, can save conservation programs time and money that can be invested elsewhere. For example, bioinformatic extraction of the mitogenome from a 1.9GB genome required 30 min, 1 CPU and 5.3GB of memory, offering a high cost‐effectiveness in terms of labour, data acquisition and analysis. As the costs associated with genome assembly decrease, a 3GB long‐read genome can cost ~$5005 in sequencing, ~$600 in labour and ~$200 in consumables (Elspeth McLennan pers. comm., 2024). Additionally, completion of a reference genome can only require 2 days of laboratory work, 6 weeks of sequencing, and 2 days for bioinformatic assembly. Although costs are not directly comparable, investment in genomic data provides a resource for a plethora of downstream applications beyond mitochondrial and eDNA (Formenti et al. 2022; de León et al. 2023; Schneider 2023; Brandies et al. 2019).

The key aspect of an eDNA assay is primer design (Figure 3: Step 4). As mentioned in step 1, primers are often developed using available reference sequences in online databases, however, regions may be missing for species or taxa (Freeland 2017; Nordstrom et al. 2022). For example, 12S, 16S, 18S sequence data is less often available compared to CO1 and CytB sequence information (Lacoursière‐Roussel et al. 2016). An advantage of a reference genome‐derived approach is that it provides researchers and managers with a complete or close to complete mitochondrial sequence. This enables the design of molecular markers for any gene in the mitogenome and provides the option to expand into nuclear marker design (Mccauley et al. 2024). *In silico*, *in vitro* and *in situ* validation methodologies (Figure 3: Step 4A‐C) should follow a standardised approach (Nordstrom et al. 2022). Since the rapid uptake of eDNA analysis, comprehensive eDNA guidelines for assay development and validation have been developed to assist researchers and managers in developing eDNA across a range of taxa and ecosystems that can be adapted to the habitat and biology of the target species (Goldberg and Strickler 2017; de Brauwer et al. 2022a, 2022b).

In summary, our methodologies and workflow for *M. georgesi* consist of four stages; (i) identifying availability of a mitogenome (Figure 3: step 1); (ii) identifying availability of a reference genome or genomic data when mitochondrial sequence does not exist in online repositories (Figure 3: step 2); (iii) bioinformatic assembly of a mitogenome from a reference genome (Figure 3: step 3a); and (iv) primer design and *in silico*, *in vitro* and *in situ* validation (Figure 3: step 4).

Our results provide *M. georgesi* managers with an eDNA assay that can be implemented into species monitoring. The assay can assist managers in resolving questions around distribution within the Bellinger River catchment, including reaches in the upper catchment and the Kalang River, and inform survey site selection through identification of occupancy hotspots. Future work is needed to evaluate efficacy of primers on Bellinger River water samples as environmental barriers such as water flow, sediment composition and microbial and enzyme activity (Barnes et al. 2014; Stoeckle et al. 2017; Stewart 2019) may influence detection. The technique will be useful for initially be used to identify areas to perform more intensive diving and trapping surveys, providing the species with a multifaceted detection and survey approach (Villacorta‐Rath et al. 2022; Lam, Sung, and Fong 2022; Nordstrom et al. 2022; Carvalho et al. 2022).

As conservation genetics moves into the genomics‐era, genomic data is becoming increasingly available for non‐model organisms, making it important to leverage and apply the information genomic resources provide. We use a reference genome‐based approach to develop an eDNA assay for *M. georgesi* . The development of species‐specific eDNA primers provides a valuable tool for managers in assessing population dynamics of this rare species, supporting informed management decisions and guiding future conservation efforts.

## Author Contributions

**Holly V. Nelson:** conceptualization (supporting), formal analysis (lead), investigation (lead), writing – original draft (lead). **Arthur Georges:** supervision (supporting), writing – review and editing (equal). **Katherine A. Farquharson:** investigation (supporting), writing – review and editing (supporting). **Elspeth A. McLennan:** investigation (supporting), methodology (supporting), supervision (supporting), writing – review and editing (supporting). **Jane L. DeGabriel:** funding acquisition (supporting), supervision (supporting), writing – review and editing (supporting). **Katherine Belov:** resources (equal), supervision (equal), writing – review and editing (supporting). **Carolyn J. Hogg:** conceptualization (lead), funding acquisition (lead), project administration (lead), resources (equal), supervision (equal), writing – review and editing (supporting).

## Ethics Statement

Collection of samples was conducted in accordance with the conditions of NSW DCCEEW Animal Ethics Committee (AEC151201‐3, AEC160503‐01 and AEC180904‐5) and Scientific Licences (MWL00102467, SL101672 and SL10255), and the Taronga Conservation Society Australia Scientific Licence SL101204.

## Conflicts of Interest

The authors declare no conflicts of interest.

## Supporting information


Data S1.

## Data Availability

The male *M. georgesi* reference genome assembly, from which the mitogenome is derived, and all raw sequencing reads including the 3‐tissue transcriptome RNA‐seq reads are available from NCBI under BioProject PRJNA1003540.
