# Supplementary material for: A Genomic‐Based Workflow for eDNA Assay Development for a Critically Endangered Turtle, Myuchelys georgesi
Source: Ecol Evol. 2025 Jan 8;15(1):e70798. doi: 10.1002/ece3.70798 (PMC11707621; doi:10.1002/ece3.70798)
Supplement: Supplementary file 1 — Data S1. [file ECE3-15-e70798-s001.docx]

## Appendix: Supplementary material

### *Myuchelys georgesi* mitogenome

The mitogenome has a base composition of 35.2% A, 26.5% C, 12% G, and 25.8% T and a GC content of 39.04%. Nine genes are encoded on the light strand including tRNA^Gln^, tRNA^Ala^, tRNA^Asn^, tRNA^Cys^, tRNA^Tyr^, tRNA^Ser^, ND6, tRNA^Glu^, and tRNA^Pro^. The remaining 28 genes are encoded on the heavy strand. A total of 7 overlapping regions with a total length of 15 bp were identified in *M. georgesi*, with the longest region of overlap (5 bp) observed between NADH dehydrogenase subunit 4L (nad4l) and NADH dehydrogenase subunit 4 (nad4). Twenty-one intergenic spacer regions with a total length of 1701 bp were observed. The longest region (1241 bp) was observed between NADH dehydrogenase subunit 5 (nad5) and NADH dehydrogenase subunit 6 (nad6) (Table S1). The total length of proteins coding genes (PCGs) was 12901 bp, representing 78.1% of the complete mitogenome. The ATG start codon initiated the majority of the PCGs, except for ATP synthase F0 subunit 6 (atp6), cytochrome c oxidase subunit III (cox3), NADH dehydrogenase subunit 4L (nad4l), and CytB that were initiated by ATA.

# Table S1. List of annotated mitochondrial genes of *M. georgesi*

| **Gene** | **Direction** | **Location** | **Size** | **Anti-codon** | **Start codon** | **Stop codon** | **Intergenic nucleotides** |
| --- | --- | --- | --- | --- | --- | --- | --- |
| trnF | + | 1-67 | 67 | GAA |  |  | 0 |
| rrnS | + | 68-1038 | 971 |  |  |  | 0 |
| trnV | + | 1039-1108 | 70 | TAC |  |  | 0 |
| rrnL | + | 1109-2720 | 1612 |  |  |  | 2 |
| trnL2 | + | 2723-2797 | 75 | TAA |  |  | 4 |
| nad1 | + | 2802-3764 | 963 |  | ATG | TAG | -1 |
| trnI | + | 3764-3836 | 73 | GAT |  |  | -1 |
| trnQ | - | 3836-3906 | 71 | TTG |  |  | -1 |
| trnM | + | 3906-3974 | 69 | CAT |  |  | 0 |
| nad2 | + | 3975-5018 | 1044 |  | ATG | TAA | 1 |
| trnW | + | 5020-5091 | 72 | TCA |  |  | 2 |
| trnA | - | 5094-5162 | 69 | TGC |  |  | 1 |
| trnN | - | 5164-5237 | 74 | GTT |  |  | 19 |
| trnC | - | 5257-5324 | 68 | GCA |  |  | 1 |
| trnY | - | 5326-5395 | 70 | GTA |  |  | 1 |
| cox1 | + | 5397-6935 | 1539 |  | ATG | TAA | 2 |
| trnS2 | - | 6938-7009 | 72 | TGA |  |  | 12 |
| trnD | + | 7023-7089 | 67 | GTC |  |  | 0 |
| cox2 | + | 7090-7776 | 687 |  | ATG | TAG | 2 |
| trnK | + | 7769-7842 | 74 | TTT |  |  | 1 |
| atp8 | + | 7844-7981 | 138 |  | ATG | AGA | 89 |
| atp6 | + | 8071-8682 | 612 |  | ATA | TAG | -2 |
| cox3 | + | 8679-9455 | 777 |  | ATA | TGA | 7 |
| trnG | + | 9463-9531 | 69 | TCC |  |  | 175 |
| nad3 | + | 9707-9874 | 168 |  | GTA | TGA | 7 |
| trnR | + | 9882-9949 | 68 | TCG |  |  | 0 |
| nad4l | + | 9950-10246 | 297 |  | ATA | TAA | -5 |
| nad4 | + | 10240-11625 | 1386 |  | ATG | TAA | -4 |
| trnH | + | 11621-11690 | 70 | GTG |  |  | 0 |
| trnS1 | + | 11691-11753 | 63 | GCT |  |  | -1 |
| trnL1 | + | 11753-11824 | 72 | TAG |  |  | 0 |
| nad5 | + | 11825-12946 | 1122 |  | ATG | ACC | 1241 |
| nad6 | - | 14188-13658 | 529 |  | ATG | TTA | 1 |
| trnE | - | 14190-14258 | 69 | TTC |  |  | 58 |
| cytB | + | 14317-15372 | 1056 |  | ATA | T(CC) | 74 |
| trnT | + | 15447-15516 | 70 | TGT |  |  | 1 |
| trnP | - | 15518-15585 | 68 | TGG |  |  |  |

Table S2. Final CO1 and CytB primer information

| **Gene** | **Sequence** | **Start** | **GC%** | **Molecular weight** | **DeltaG** |
| --- | --- | --- | --- | --- | --- |
| CO1 | *Forward* |  |  |  |  |
|  | acattggcaccctctacctg | 41 | 55 | 6012.9 g/mole | -3.14 kcal/mol |
|  | *Reverse* |  |  |  |  |
|  | aattaaggcgtgggctgtaa | 192 | 45 | 6221.1 g/mole | 5.36 kcal/mol |
| CytB | *Forward* |  |  |  |  |
|  | aatctcccacatccaacgag | 186 | 50 | 6015 g/mole | -3.61 k/mol |
|  | *Reverse* |  |  |  |  |
|  | atgcggtggctatgactagg | 373 | 55 | 6213.1 g/mole | -4.16 k/mol |

Table S3. *In vitro* validation tissue specimens

| Specimen ID | Species | Sample ID | Year | Type | Location sampled |
| --- | --- | --- | --- | --- | --- |
| C10031 | *Myuchelys georgesi* | MG_heart147B | 2021 | Heart | Taronga Zoo |
| UC<Aus>AA063727 | *Emydura macquarii* | AA063727 | 2015 | Skin | Bellinger River |

Table S4. *In situ* validation samples

| Source | Tank size | Species | N | Samples |
| --- | --- | --- | --- | --- |
| Tank 1 | 4000 L | Bellinger River Turtle *(Myuchelys georgesi)* | 5 | 2 x 500 mL (1 L) |
| Tank 2 | 4000 L | Bellinger River Turtle *(Myuchelys georgesi)* | 5 | 2 x 500 mL (1 L) |
| Tank 3 | 4000 L | Bellinger River Turtle *(Myuchelys georgesi)* | 5 | 2 x 500 mL (1 L) |
| Pond (control) | 2000 L | Murray River Turtle *(Emydura macquarii)*  Eastern long-necked Turtle *(Chelodina longicollis)* | 4  2 | 2 x 500 mL (1 L) |

Figure S1. Complete nucleotide sequence of Cytochrome Oxidase 1 gene output by MitoHiFi and used as input into Primer3Plus. Forward and reverse primer pairs are highlighted in grey.

**>CO1**

tattaaaccgctgactattttctactaaccataaagacattggcaccctctacctgatttttggggcctgagcaggaataatcggaacagctcttagtctattaattcgaacagaactaagccaaccaggtcccctattaggagatgatcaagtatacaatgtaattgttacagcccacgccttaattataatctttttcatagttatacctattataattggtggatttggaaactgattagttccaataataattggatcgccagatatagcatttccacgaatgaataatataagcttttgacttctaccaccatcactactactacttctagcctcctctggtatcgaagccggagctggaacagggtgaactgtatatccccctttggctggaaatatagcccacgccggagcttctgtcgacctaactatcttctccctacacttagccggggcgtcctcaattttaggggccatcaacttcatcaccaccgcaattaatataaaaaccccatcaatatcacaataccaaacaccacttttcgtatgatcagtacttatcacagctgtattactattactctcccttccagtacttgctgcaggcattactatacttttaacagaccgaaacctaaatacaaccttctttgatccatctggtggaggagacccaatcctataccaacacctattttgattctttggccaccccgaagtatacattctcatccttcccggcttcggaataatttctcacgtcgttgcttattatactggtaaaaaagaaccattcgggtacatgggaatagtctgagcaataatatcaatcggattcttaggattcatcgtttgagctcaccatatattcacagtaggaatagacgtagacacccgagcctactttacatcagcaacaataatcattgctatccccacaggtgtcaaagtatttagctgactagccaccctccacggaggaataattaaatgagatgcccctatactatgagctctgggatttatttttctatttaccattggaggactaacaggtattgtattagctaactcatcactagatatcgtactacacgatacatactatgtagtagcacacttccattatgtactatcaatgggggccgtatttgccattatagccggatttacccactgattcccattatttacaggattctcactaaaccaaacatgggcaaaactacaattcgtagtaatattctttggcgtaaacataacattcttcccacaacacttcctaggtttagctggtataccccgacgatactcagattatccagacgcctacacaatatgaaactccatctcatcaattggatcaataatctccatagcagcagtaatcataatactagtcattatctgagaggccttctcatcaaagcgaaaaatagtactgattgaaccacccctaatcaacgtagaatgactaagcggttgtccgccatccagtcacacctatgaagaatccgcacatatattataa

Figure S2. Complete nucleotide sequence of Cytochrome B gene output by MitoHiFi and used as input into Primer3Plus. Forward and reverse primer pairs are highlighted in grey.

**>CytB**

ataaaaaacacaaatccactattaaaaattattaacaacaccttcatcgatctccccaccccctccaacatctccgccctatgaaacttcggatcactactaggaatatgcctaattctacaactagccacaggaatcttcctagctatacactactcgcccgatatctccatagcattctcatcaatctcccacatccaacgagacgtccaatatggttgactgattcgaaacatacacgccaacggtgcttcactatttttcatatgcatttacctccatattggacgtggaatctactacggttcctacctctacaataaaacctgaaacactggagtaattctactattcctagtcatagccaccgcattcgtgggctacgtactaccatgaggccaaatatcattctgaggggctacagtaatcaccaaccttctatcagccatcccatatgcaggccctacacttgtagaatgaatctgaggaggattctccgtagacaacgccaccctgactcgattcttcacattccactttctaatcccattcgctatcctaggaataaccatactacacctcctactactacacgaaacaggatcaaacaacccaacaggattaaactcaaactgcgacaagatcccattccacccatatttttcttacaaagacctactaggcctcatcctaataatcatatgcctgctcaccctcaccctattttacccaaacctactaggagatccagacaacttcacaccagccaacccactaaccaccccaccacacatcaaaccagagtgatacttcctattcgcttacacaatcctacgttcaatccccaacaaactaggtggcgtcctagccctattcatatcaatcctagtactactaaccataccaatactccacctatcaaaacaacgaacaaccacattccgaccaatagcacaaatcctcttctgatgcttaaccactgacctactaatcctaacatgaatcggaggccaaccagtagaagacccattcatcctcattggacaaatcgcctccctactatacttcaccatcatcttcatcatcacacctataacaagcttaattgaaaacaaaatactaaaccaat
